# Knotted Nasogastric Tube Removed With a Bronchoscope

**DOI:** 10.7759/cureus.40896

**Published:** 2023-06-24

**Authors:** Nicholas R Munoz, Armin Hojjat, Galvin Dhaliwal, Ajay Adial

**Affiliations:** 1 Internal Medicine, Temecula Valley Hospital, Temecula, USA; 2 Pulmonology and Critical Care, Temecula Valley Hospital, Temecula, USA

**Keywords:** spontaneous knot formation, pull out resistance, gastric tube, ng, complication, nasogastric tube complications, knot nasogastric, bronchoscope, nasogastric tube (ngt), nasogastric tube

## Abstract

Nasogastric (NG) tube insertion is a routine procedure performed for a variety of indications, such as delivering enteral nutrition. NG tubes can be associated with complications, including knotting of the tube. The case of a 68-year-old who was admitted to the hospital for AIDS complicated by septic shock is presented. The patient received an NG tube to provide enteral nutrition, which was subsequently found to be clogged. An X-ray of the pharynx revealed a knot at the distal end of the NG tube. The knotted NG tube was removed with a fiberoptic bronchoscope through the nostril. The knotting of an NG tube is a rare complication. Clinicians should be aware of alternative methods of removing knotted NG tubes, including the use of a fiberoptic bronchoscope.

## Introduction

Nasogastric (NG) tube insertion is a common procedure for hospitalized patients. Indications for NG tube insertion include administering medications, administering nutrition, decompressing the stomach in bowel obstructions, and diagnosing upper gastrointestinal bleeds. Contraindications of NG tubes include skull fractures, facial trauma, esophageal trauma, esophageal obstruction, or abnormal anatomy [1]. Complication rates from NG tube placement vary from 0.3% to 8% and include pneumothorax, pulmonary hemorrhage, tube knotting, impaction, esophageal perforation, and intracranial entry [2]. The case of a 68-year-old with a history of HIV with a hospital course complicated by a knotted NG tube is presented. The patient had an NG tube inserted for delivery of enteral nutrition, as he was malnourished with inadequate oral intake. Following placement, the NG tube was found to be clogged. After the tube was unable to be removed by pulling, an X-ray showed the NG tube was knotted. A fiber optic bronchoscope was used to remove the tube from the pharynx. Although complication rates are low, clinicians should be able to treat complications of NG tubes.

## Case presentation

The patient was 68 years old with a past medical history of HIV and narcolepsy admitted to the hospital for weakness, altered mental status, and shortness of breath. He was diagnosed with HIV 30 years previously and admitted to being non-compliant with HIV medication for 15 years. He was found to be HIV positive with a CD4 count of 86 indicating AIDS. The patient was found to be obtunded with a Glasgow Coma Scale score of 10. He had bilateral rhonchi on lung auscultation and poor dentition. He was initially hypoxic with evidence of pneumonia in the form of patchy bilateral infiltrates on a chest X-ray. He went into septic shock, was placed on broad-spectrum antibiotics, upgraded to ICU level of care, and started on a norepinephrine drip. He received a bronchoscopy with bronchoalveolar lavage and was found to have *Pneumocystis jirovecii* pneumonia. He improved while on targeted antibiotics. Norepinephrine was weaned, and he was downgraded from the ICU to a lower level of care.

**Figure 1:**
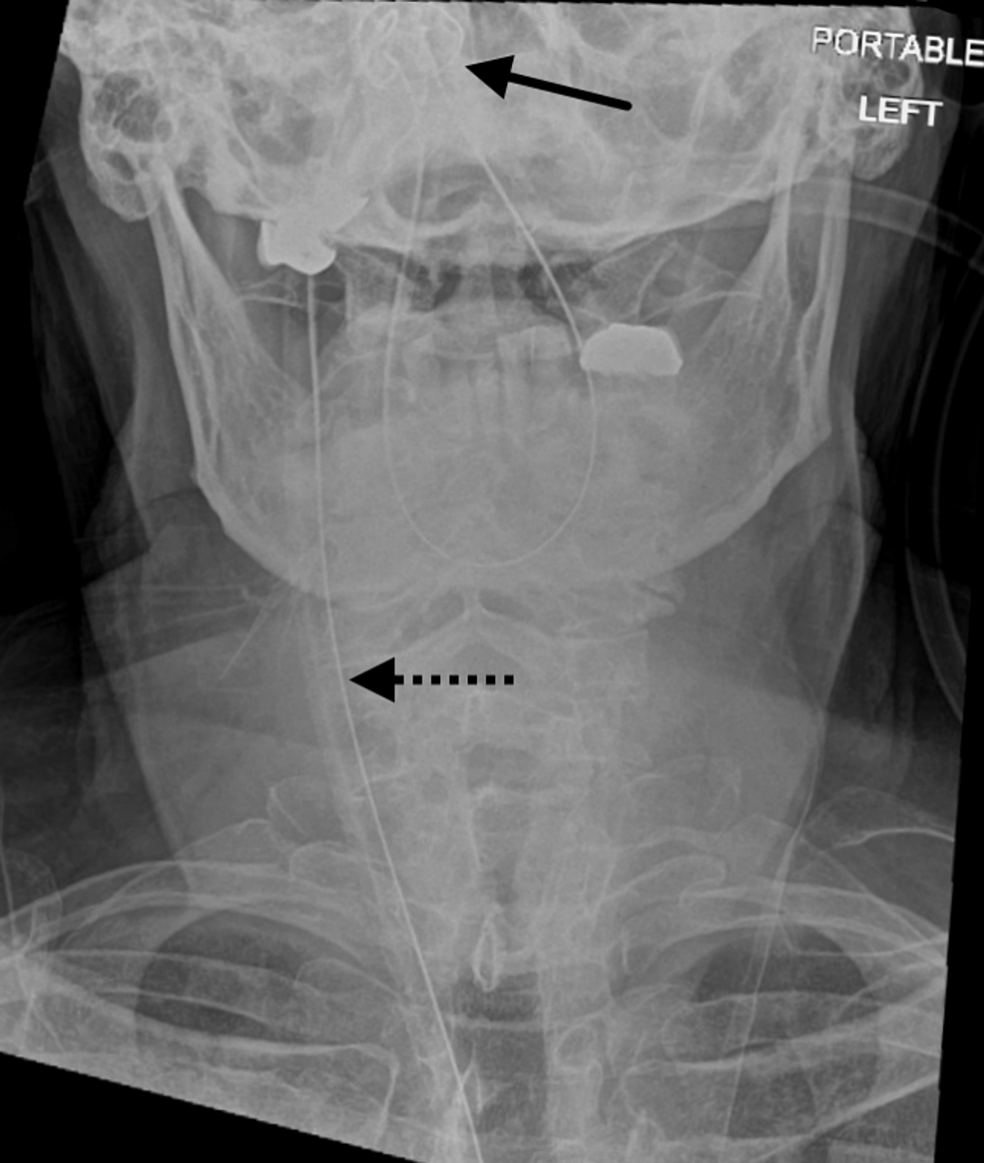
X-ray of a knotted nasogastric (NG) tube in the patient's pharynx. The solid arrow indicates the knot in the NG tube, while the dotted arrow points to the proximal NG tube located outside the body.

**Figure 2:**
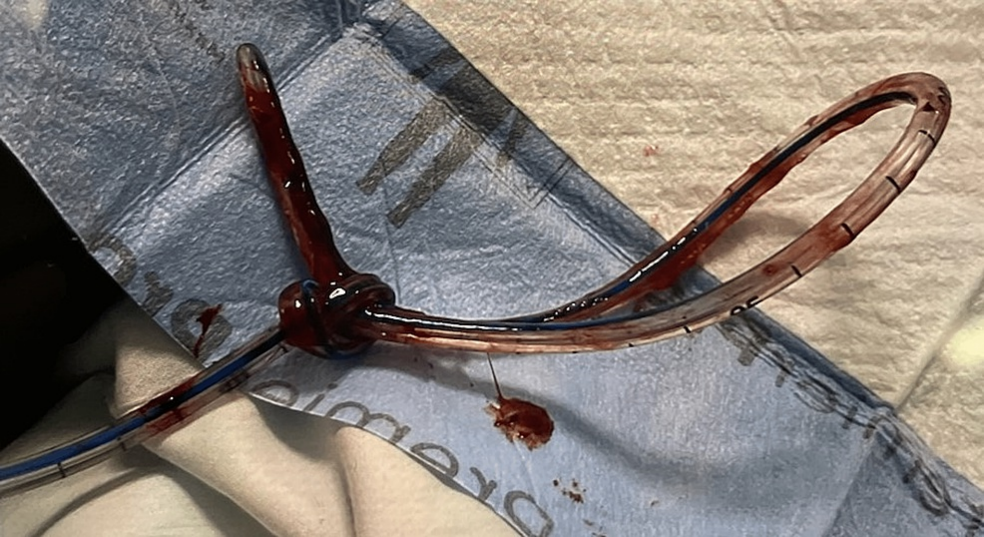
The knotted nasogastric tube found in the patient's pharynx, which was removed through the right nostril with a bronchoscope.

While the patient was recovering from sepsis, he was found to have decreased oral intake and malnutrition. A speech therapist evaluated the patient and found he had oropharyngeal dysphagia. An NG tube was placed for enteral nutrition, as the patient was unable to safely swallow. During NG tube insertion, he was noncompliant with swallowing on command and was moving his head. Shortly after insertion, the NG tube was found to be clogged, as the liquid was unable to be passed through it. X-ray of the head and neck showed a knotted NG tube in the pharynx (Figure 1). Attempts to remove the NG tube by hand were met with resistance. The pulmonology team was consulted, and they decided to remove the tube with a fiber-optic bronchoscope. The NG tube was found in the hypopharynx, it was removed by bronchoscope intact through the right nostril (Figure 2). Removal with a bronchoscope was preferred over cutting the tube and removing through the mouth as the patient was non-compliant with instructions to hold still, had poor dentition, and the tube was unable to be visualized through the oral cavity. The patient recovered well after NG tube removal. He received a percutaneous endoscopic gastrostomy tube for tube feeds as he continued to have dysphasia. He was medically stable and discharged home on hospice.

## Discussion

NG tube placement for enteral nutrition is a routine aspect of care for many hospitalized patients. A total of 1.2 million feeding tubes are placed annually in the United States as 20-50% of hospitalized patients are recognized to be malnourished [3,4]. Enteral feeding is preferred as parenteral nutrition is associated with gut mucosal atrophy, electrolyte imbalances, and bloodstream infections [5-7]. NG tubes are usually placed blindly through the nose into the stomach, although endoscopy or fluoroscopy can be used for difficult-to-place NG tubes [8]. After NG tube insertion, radiographic confirmation is the most accurate method of confirming correct placement [9].

The management of NG tubes includes irrigating the tube before and after every feed or medication administration to ensure patency [10]. The discomfort of the nasal passage is common after NG tube insertion and may be managed with a local anesthetic spray [11]. Pressure ulcers in the nasal cavity may result if an NG tube is too large or secured improperly. In patients who are unconscious or otherwise at high risk of nasal pressure ulcers, intermittent retaping of the tube is indicated to decrease pressure points [12]. As an NG tube interrupts the normal functioning of the lower esophageal sphincter, the development of acid reflux or chest pain may herald esophagitis, and removal of the tube is indicated [13].

NG tubes should be removed as soon as possible to minimize the risk of complications. If there is resistance to manual pulling, removal should be halted, and imaging obtained [14]. If imaging reveals a knotted NG tube that is unable to be removed by pulling, alternative methods should be considered, including removing the tube through the mouth using forceps, cutting the tube and removing the knot through the mouth, passing a nasopharyngeal tube over the NG tube, and removing both, or removing the tube with a fiberoptic bronchoscope, as in this case [15-17]. The location of the knot must be taken into consideration when planning the removal of an NG tube. For example, if the knot is in the esophagus, a flexible endoscope would be a more appropriate tool compared to a fiberoptic bronchoscope. Different mechanisms have been proposed for NG tube knot formation, including small stomachs, excessive manipulation of the tube, narrow tubes, deep insertion, and using a tube that is too long [18,19].

In the case presented, the patient's excessive movements and manipulation of the tube likely caused the line to loop into a knot. Clinicians should be aware of the knotting of an NG tube as a possible complication. To avoid knotting of the NG tube, the patient should be kept still, and excessive manipulation of the tube should be avoided. Care should be taken to choose the correct tube size and depth of insertion. When pulling by hand alone is insufficient to remove a knotted NG tube, alternatives should be considered such as removal by fiberoptic bronchoscope.

## Conclusions

NG tube insertion is a routine procedure for many hospitalized patients. Due to the widespread use of NG tubes in hospitals, complications such as knotting of the tube can occur. Clinicians should be aware of different methods to remove knotted NG tubes if regular removal techniques, such as pulling by hand, fail. The case presents a new method of removing a knotted NG tube using a fiberoptic bronchoscope. If a knotted NG tube is unable to be removed by pulling alone, removal with a fiberoptic bronchoscope should be considered. In the future, prospective studies would be useful to evaluate methods of knotted NG tube removal.
